# Nanomechanical mapping helps explain differences in outcomes of eye microsurgery: A comparative study of macular pathologies

**DOI:** 10.1371/journal.pone.0220571

**Published:** 2019-08-07

**Authors:** Gabriele Ciasca, Valeria Pagliei, Eleonora Minelli, Francesca Palermo, Matteo Nardini, Valentina Pastore, Massimiliano Papi, Aldo Caporossi, Marco De Spirito, Angelo Maria Minnella

**Affiliations:** 1 Fondazione Policlinico A. Gemelli IRCCS, Roma, Italia; 2 Istituto di Fisica, Università Cattolica del Sacro Cuore, Roma, Italia; 3 Istituto di Oftalmologia, Università Cattolica del Sacro Cuore, Roma, Italia; 4 Department of Medical Science, Neuroscience and Sense Organs, Eye Clinic, University of Bari “A. Moro”, Bari, Italy; University of Manchester, UNITED KINGDOM

## Abstract

Many ocular diseases are associated with an alteration of the mechanical and the material properties of the eye. These mechanically-related diseases include macular hole and pucker, two ocular conditions due to the presence of abnormal physical tractions acting on the retina. A complete relief of these tractions can be obtained through a challenging microsurgical procedure, which requires the mechanical peeling of the internal limiting membrane of the retina (ILM). In this paper, we provide the first comparative study of the nanoscale morphological and mechanical properties of the ILM in macular hole and macular pucker. Our nanoscale elastic measurements unveil a different bio-mechanical response of the ILM in the two pathologies, which correlates well to significant differences occurring during microsurgery. The results here presented pave the way to the development of novel dedicated microsurgical protocols based on the material ILM properties in macular hole or pucker. Moreover, they contribute to clarify why, despite a common aetiology, a patient might develop one disease or the other, an issue which is still debated in literature.

## Introduction

In the last two decades, a large body of evidence has emerged that physical forces are critical regulators in biology as well as fundamental players in the development of many diseases [1–4]. An exceptionally wide range of clinical problems that bring patients to the doctor’s office are indeed connected to an alteration of the mechanical homeostasis in tissues and organs [5, 6]. Many ocular diseases are listed among these pathologies, including glaucoma, myopia, ocular trauma and other clinical conditions such as macular pathologies [7, 8]. The eye itself has indeed a complex biomechanical behavior [9] and many structures that are crucial for the visual function are subjected to significant physical forces due to the intraocular pressure, intraocular and extra-orbital muscles, and external forces on the eye [7]. The mechanical homeostasis of the eye is also affected by a wide range of age-related phenomena involving connective tissue remodeling. An example is the posterior vitreous detachment (PVD), a physiologically-occurring separation between the posterior vitreous cortex and the internal limiting membrane (ILM) of the retina [10]. In pathological conditions, an incomplete PVD exerts vitreomacular tractions (VMT) on the macula that are associated with a multitude of clinical conditions causing a decreased visual activity (VA). These include macular pucker, macular hole, and vitreo-macular traction syndrome [11]. Forces are exerted between the posterior vitreous cortex and the inner limiting membrane (ILM) of the retina, the ultimate site of pathologic tractions at the vitreoretinal interface [10].

ILM belongs to the class of the basement membranes (BMs), thin sheets of extracellular matrix (ECM) at the basal side of every epithelium; specifically, it is the BM of Müller cells and its molecular constituents include members of the laminin, nidogen, collagen IV, and proteoglycan families, the latter contributing to the elevated ILM hydration in-vivo [12, 13]. The ultrastructural features of BMs have been studied mainly by Transmission Electron Microscopy, showing a bi-functional organization of the membrane with the two outer surfaces greatly differing in their capacity to promote cell adhesion [13]. Significant differences between the two membrane sides are also measured from a morphological point of view; the anterior (vitreal) side of the ILM is usually smooth, while the posterior (retinal) side is markedly irregular [11, 14, 15]. Vitrectomy with the removal of the ILM is a valuable therapeutic option to obtain a complete relief of pathological vitreomacular tractions. However, surgical peeling is an exceptionally complex procedure as the ILM is extremely thin (a few microns in thickness in its hydrated state), transparent and in close proximity to the highly delicate retinal structures that are easily undergoing severe damages [10, 11, 16]. The safety and the effectiveness of this procedure depends, on the one hand, on how well the thin and transparent ILM can be identified, on the other hand, on the peculiar ILM material properties such as thickness, stiffness and elasticity, which largely determine the adhesive interaction between the surgical instruments and the membrane, and influence the ILM peeling behavior. While the former problem was addressed thanks to the introduction of vital dyes in vitreoretinal surgery [10], the latter one requires a better understanding and a proper instrumentalization of the biomechanical properties of the ILM such as micromorphology, thickness and stiffness distribution [12, 14]. A careful characterization of the ILM material properties in different clinical conditions appears even more relevant if one considers that vitreomacular tractions are associated with a broad range of maculopathies, and the reason why patients may develop, for example, a macular hole rather than a macular pucker remains unclear [17]. In this paper, we provide a comparative study of brilliant blue G (BBG) assisted peeling of the ILM for the surgical treatment of macular hole (MH) and macular pucker (MP). We point out and quantify differences between the two pathologies occurring during vitrectomy, which hint at different material, mechanical and adhesion properties of the peeled membrane. Then we provide the first direct comparison between the morphological and nanomechanical properties of surgically removed ILM samples obtained from patients diagnosed with MH and MP. To this purpose, we used the Atomic Force Microscopy (AFM), a powerful biophysical technique that allows to image and quantitatively investigate the morphological and mechanical properties of biological samples at the nanoscale level in nearly physiological environment [18–24]. Our high-resolution elasticity maps allowed us to highlight a different biomechanical behavior of samples obtained from patients diagnosed with the two different pathologies, contributing to add another piece of understanding on the reason why these two pathologies, although shearing a common aetiology, result in a different clinical outcome.

## Materials and methods

The study was approved by the Institutional Review Board of Fondazione Policlinico Universitario Agostino Gemelli IRCCS and Università Cattolica del Sacro Cuore (Prot. 18070/17 ID:1579) and conducted in accordance with the ethical guidelines. A total of 24 patients, 13 men and 11 women, aged from 59 to 82 years (mean age of 71 years) were recruited for the study. Patient recruitment was carried out with informed consent. Among the recruited patients, 9 were diagnosed with macular hole and 15 with macular pucker. All the 9 cases of macular hole were full thickness (FTMH) and idiopathic. Among the eyes with macular pucker, only 1 case was associated with branch retinal vein occlusion (BRVO).

The study included a series of 24 eyes belonging to the 24 enrolled patients undergoing pars-plana vitrectomy and ILM peeling. Samples were obtained from the recoloring of the ILM with Brilliant Blue G, after the removal of ERM using Triamcinolone acetonide.

Prior to AFM examinations, peeled samples were further observed under the microscope. This evaluation allowed us to distinguish between sample regions showing the presence of ERM remnants and those consisting of ILM alone. The latter were used in the following analysis. In testing the vitreous and the retinal side of the membranes, we took different flaps.

Mechanical measurements were performed immediately after surgical peeling, keeping ILM in its hydrated state, using JPK Nanowizard II Atomic Force Microscope. Prior to measurements, ILM specimens were immobilized on a polylysine covered plastic Petri dish. All preparative steps were performed in a sterile PBS buffer and mechanical manipulations were kept minimal. Measurements were performed at room temperature using Mikro-Mash silicon cantilevers with a tip radius of about 10 nm and spring constant of approximately 0.05 N/m (CSC37, Mikro-Mash). The cantilever spring constant was carefully determined before each measurement by thermal calibration and the measured values were checked using polymeric scaffold of known stiffness. Force curves were acquired using an indentation force of 5 nN at different scanner velocity during indentation in the range 1–35 *μ*m/s, with the aim to investigate the viscoelastic response of samples. Indentation curves were analyzed using the Sneddon model:

$$\begin{array}{c} F\left(\delta \right)=\frac{2E\tan(\alpha )}{\pi (1-\nu^{2})}\delta^{2} \end{array}$$ (1)

where *E* stands for the apparent Young’s modulus, *ν* for the Poisson ratio and *δ* for the indentation depth. The Poisson ratio was set at 0.5 to account for the material incompressibility. The *apparent Young’s modulus* term (referred to as *E* in the following) accounts for the fact that *E* depends both on the scanner velocity during indentation and on the indentation depth.

Since biological samples are characterized by spatial heterogeneity, their physical and functional properties are better described by nano-mapping rather than randomly located single point measurements [25–27]. The local biomechanical response of ILM samples was investigated mapping *E* retrieved using Eq 1, according to [28–34]. The Map size was 20 *μ*m × 20 *μ*m. Different resolutions were adopted, depending on scanner velocity. 32 × 32 pixels’ maps, which correspond to a spatial resolution of 625 nm, were acquired for a scanner velocity of 5 *μ*m/s. 8 × 8 pixels’ maps (i.e. 2.5 *μ*m spatial resolution) were acquired for other scanner velocities. Typical acquisition times were of the order of a few tens of minutes, for 32 × 32 pixels’ maps. Lower/higher scanner velocity requires lower/higher acquisition time. The role played by viscous forces in the biomechanical response of tissues was qualitatively evaluated studying the dependence of *E* on the scanner velocity during indentation. A quantitative evaluation of the contribution of dissipative forces has been obtained estimating the energy dissipated during indentation (or Hysteresis *H*). *H* was computed as the difference between the area (*A*_*E*_) under the approach curve (*F*_*E*_(*δ*)) and that (*A*_*R*_) under the retract curve (*F*_*R*_(*δ*)) normalized by *A*_*E*_:

$$\begin{array}{c} H=\frac{\int_{0}^{\delta }F_{E}\left(\delta \right)d\delta -\int_{0}^{\delta }F_{R}\left(\delta \right)d\delta }{\int_{0}^{\delta }F_{E}\left(\delta \right)d\delta }=\frac{A_{E}-A_{R}}{A_{E}} \end{array}$$ (2)

Tissue topography of the hydrated membrane was evaluated in liquid environment mapping the tip contact point obtained with Eq 1. Subsequently, the same samples were dehydrated in ethanol solutions to investigate the nanoscale sample topography in AFM contact-mode, acquiring maps of 20 *μ*m × 20 *μ*m and 512 × 512 pixels. The RMS (root mean square) sample roughness was evaluated using the JPK dataProcessing software according to the equation:

$$\begin{array}{c} R_{rms}=\sqrt{\sum_{i=1}^{n}\frac{{\left(z_{i}-z_{m}\right)}^{2}}{N-1}} \end{array}$$ (3)

where *N* is the number of data points; *z*_*i*_ is the height of *i*-th point, and *z*_*m*_ is the mean height [35]. A total of approximately 90 ILM specimens obtained from 24 eyes were analyzed. Tissue maps were plotted using the software OriginPro 8.5 (Microcal). This software uses thin plates spline (TPS) algorithm to smooth map data. All individual stiffness and hysteresis values for each specimen were summarized in OriginPro 8.5 (Microcal) to obtain the normalized frequency distribution of stiffness values. All average data are given as mean ± standard deviation of the mean, unless otherwise specified. The statistical significance of differences in mean values was assessed with the Welch’s t-test in Excel 2010 (statistical significance was set at *P* ≤ 0.05). Microscope operating views of the ILM peeling were quantitatively analyzed using ImageJ, in which the Segmentation Editor package has been previously installed. In the main menu, in particular, selection brush was enabled with a size of 20 pixels.

## 1 Results

### 1.1 Differences occurring during ILM peeling hint to different membrane material properties in macular hole and macular pucker

**Fig 1:**
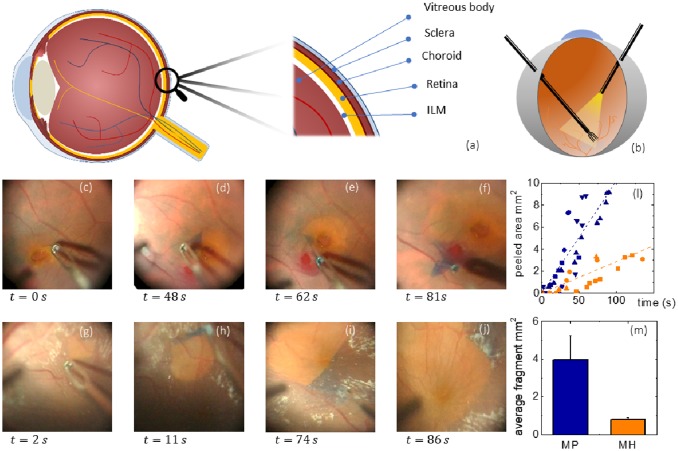
Schematic view of a human eye highlighting the location of the inner limiting membrane (ILM) (a). Schematic view of the surgical set-up used during ILM peeling (b). Microscope operating view of the ILM peeling for a representative macular hole (MH) (c-f) and macular pucker (MP) (g-j). Peeled area over time for MP (orange) and MH (blue) in different vitrectomies (l). Average area of peeled fragments in MH (orange) and MP (blue) (m).

Surgical peeling of the ILM in macular hole (MH) and pucker (MP) is considered a technically challenging procedure even for expert surgeons, because of the difficulty in visualizing the membrane, initiating the peel and properly determining its extent and completeness. This is largely due to the extremely thin nature of this membrane and its proximity to the retinal surface, which can potentially undergo severe damages during the surgery. ILM indeed is few microns thick in its hydrated conditions and constitutes the boundary within retina and vitreous body, as schematically shown in Fig 1a. The contrast between ILM and the underlying retinal structures can be enhanced using vital dyes such as Brilliant Blue G (BBG), which has been adopted in the present study. The surgical set-up is schematically described in Fig 1b. Instrumentation is constituted of a light pipe and grasping forceps. Both are inserted from the anterior part of the eye and are hold through a precision grasp, to improve precision in handling it. A viewing system is positioned in the front of the anterior part of the eye to allow the surgeon to see the internal part where instruments are inserted. The optical system is also used to record movies and acquire snapshots of operating views (Fig 1(c)–1(j)). During surgery, retina is illuminated with a light pipe, a part of the membrane near fovea is elevated and grasped with gripping forceps, which are then lifted to separate the membrane from the retina. After elastic tolerance is exceeded, it begins to tear (an example is shown in Fig 1(c)–1(e)). In Fig 1(c)–1(f) and 1(g)–1(j) we show a microscope operating view of the ILM peeling for a representative MH and MP vitrectomy, respectively. Different snapshots were acquired at different times during surgery showing the grasping of the ILM flap at *t* = 0s, and the subsequent progression of the vitrectomy. Fig 1c and 1g show the initial phase of the peeling for MH and MP, respectively. The contrast between the BBG-stained ILM and the underlying unstained retinal surface allows us to monitor precisely the extent of the peeled area for the two pathologies (Fig 1(d)–1(f) and 1(h)–1(j)). A qualitative observation of Fig 1(c)–1(j) hints at two possible differences between MH and MP: at first, it seems that larger areas are removed in a minor time in MP vitrectomy in comparison to MH; secondly, we notice two ways of tearing, one in MH, which breaks the membrane in smaller pieces (Fig 1j and 1h), the other in MP, which produces larger pieces of ILM. To assess these differences, we measure the peeled area over time during different vitrectomies for both MH (Fig 1l, orange) and MP (Fig 1l, blue) using the software package ImageJ. Different symbols are used to indicate different patients. A linear trend was fitted to data of different patients with either MP or MH, obtaining an average peeled area of 0.106 ± 0.003 mm^2^/s for MP and 0.030 ± 0.002 mm^2^/s. During MP surgery larger areas are removed in less time than during MH intervention. Moreover, we quantified the average area of fragments occurring during ILM peeling in MP and MH interventions, showing that larger fragments of ILM are usually produced during MP rather than MH (Fig 1m). We measured an average fragment area of 4.1 ± 1.3 mm^2^ for MP and 0.83 ± 0.11 mm^2^ for MH.

These differences occurring during ILM peeling might be due to different structural, morphological or mechanical properties of the membrane in the two pathologies, which are investigated in the following using AFM.

### 1.2 Nanoscale mechanical characterization of the ILM unveils that the retinal side of the membrane is significantly stiffer in macular hole than in macular pucker

**Fig 2:**
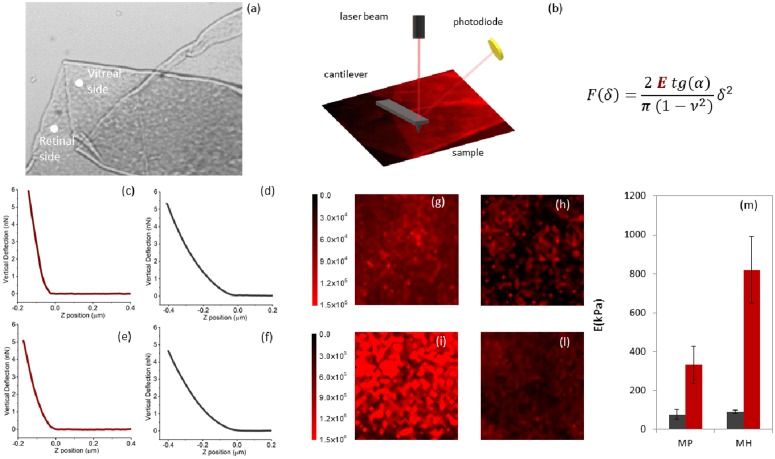
Optical micrograph of an inner limiting membrane (ILM) specimen surgically removed from a patient diagnosed with a macular pucker (a). Schematic view of the experimental setting during an atomic force microscopy (AFM) experiment (b). Four typical force distance curves acquired on the retinal and vitreal side of membranes extracted from patients affected by macular pucker (c-d) and macular hole (e-f). Four representative *E*-maps of the retinal (g,i) and vitreal side (h,l) of two ILM samples obtained from a patient diagnosed with macular hole and pucker. Bar plot of the average *E* values calculated for all the recruited patients diagnosed with macular hole and pucker for both ILM sides.

The mechanical response of ILM samples was investigated through the acquisition of AFM force-distance (FD) cycles that can be mathematically analyzed to obtain the apparent Young’s modulus (*E*) and the AFM hysteresis (*H*). The former provides information on the sample stiffness, the latter quantitatively evaluates the contribution of dissipative forces. In Fig 2(c)–2(f), we show four typical force distance cycles, acquired on the retinal and vitreal side of membranes from patients affected by macular pucker (c-d) and macular hole (d-e), together with an optical micrograph of a surgically removed ILM specimen (Fig 2a) and a sketch of the experimental setting (Fig 2b). Measures were carried out directly in a liquid environment in order to minimize mechanical modifications of the sample with respect to its hydrated form. The curves appear to be qualitatively different, suggesting a difference in the mechanical properties of the two membrane sides. Biological tissues, ILM included, are usually characterized by an extremely complex structural organization; therefore single point measurements, like those shown in Fig 2, are usually not representative of the whole tissue. Thus, we acquired different FD curves at different positions over the sample surface to obtain an elasticity map of the scanned area (lateral size 20 *μ*m × 20 *μ*m and 32 × 32 pixels). This better describes the heterogeneous behavior of the samples at the nanoscale level.

The Young’s modulus *E* was retrieved analyzing each FD approach curve with the Sneddon model for conical indenters (Eq 1). In Fig 2(g)–2(l) we report four representative *E*-maps of the retinal (g,i) and vitreal side (h,l) of two ILM samples obtained from a patient diagnosed with macular hole and pucker, respectively. A qualitative analysis of Fig 2(g)–2(l) suggests that the vitreal side of the ILM has a similar elastic response in the two pathological states. Interestingly, the retinal side appears to be significantly stiffer in the case of macular hole than macular pucker. In Fig 2m, we report the average *E* values calculated for all the recruited patients diagnosed with macular hole and pucker, separately. Data are provided as mean ± SEM. We obtained (78 ± 24) kPa and (90 ± 8) kPa for the vitreal side of the macular pucker and hole, respectively, and (332 ± 95) kPa and (819 ± 172) kPa for the retinal side (Table 1). According to these findings, the retinal side appears to be significantly stiffer in macular hole than in pucker; conversely, significant differences between the vitreal sides were not detected. This result is highly interesting as it unveils a difference in the material properties of the ILM that might be correlated with the different outcome of the two pathologies. In this regard, it is likely that different rigidity of the ILM in the two cases may alter the mechano-transduction of vitreomacular tractions at the retinal level.

**Table 1:** Mechanical properties of internal limiting membrane. Average Young’s modulus, average hysteresis and RMS of retinal and vitreal side for macular pucker patients in the first row and macular hole in the second row.

	*E*_*R*_ (kPa)	*E*_*V*_ (kPa)	*H*_*R*_ (a.u)	*H*_*V*_ (a.u.)	*RMS*_*R*_ (nm)	*RMS*_*V*_ (nm)
MP	332 ± 95	78 ± 24	0.48 ± 0.04	0.39 ± 0.03	83 ± 13	43 ± 3
MH	819 ± 172	90 ± 8	0.49 ± 0.03	0.29 ± 0.05	67 ± 6	36 ± 4

Data are reported as mean±SEM

### 1.3 Dissipative forces play a relevant role in the mechanical response of internal limiting membrane

**Fig 3:**
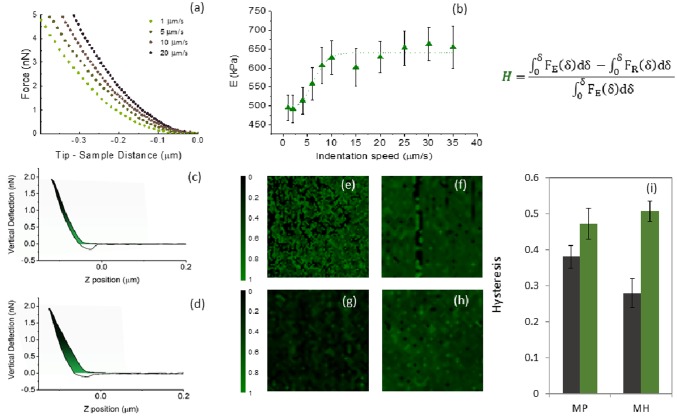
Representative set of force-distance curves performed at different scanner velocities during indentation in the range 1–20 *μ*m/s, acquired in the same position on the retinal side of an inner limiting membrane (ILM) sample obtained from a macular hole (MH) patient (a). Average Young’s Modulus (*E*) value as a function of the scanner velocity during indentation (b). Data are provided as mean ± SEM. Four representative hysteresis maps acquired on the vitreal side (e, g) and the retinal side (f,h) of a patient diagnosed with a macular pucker and a macular hole, respectively. Map size is 20 *μ*m × 20 *μ*m. Bar plot of the mean Hysteresis (*H*) value for the vitreal (orange) and retinal (blue) measured on all the investigated samples in the case of macular hole and pucker (i).

In most of the AFM studies, the basic assumption is that ILM behaves like an elastic body: hence, dissipative forces are neglected, and the Young’s modulus is treated as unaffected by probe dynamics. We checked whether this assumption is verified in Fig 3a acquiring a representative set of force-distance curves performed at different scanner velocities during indentation in the range 1–20 *μ*m/s. Curves were acquired at the same position on the retinal side of an ILM sample obtained from a patient diagnosed with a macular hole. Increasing the indentation rate, a corresponding increase in the reaction force is observed. This evidence suggests that the internal limiting membrane does not behave as a pure elastic body; conversely, dissipative forces play a relevant role in determining its mechanical response. Given the heterogeneous and local nature of the mechanical response of ILM shown in Fig 3a, we acquired several 8 × 8 Young’s modulus maps at different scanner velocities during indentation in the range 1–35 *μ*m/s.

In Fig 3b we reported the average *E* value computed from each map as a function of the scanner velocity for the vitreal side of a representative ILM sample. Data are provided as mean ± SEM. Increasing the scanner velocity, we observe a monotonous increase of *E* up to a plateau value at approximately 15 *μ*m/s. The behaviour of the average *E* as a function of the scanner velocity during indentation shows a qualitative good agreement with the standard linear solid (SLS) mode. This model consists of two systems in parallel; the first, referred to as the Maxwell arm, contains a spring (*K*) and dashpot (viscosity *η*) in series, the other system contains only a spring *k*_*e*_. As demonstrated in [36], the sample reaction force *F*(*z*) can be expressed, under the boundary condition *F*(*z* = 0) = 0 and the assumption of constant indentation velocity and as *F*(*z*) = *k*_*e*_*z* + *ην* − *ηνe*^(−*Kz*/*ην*)^. According to this equation, the sample reaction force at very low velocity is dominated by pure elastic contribution *F*(*ν* → 0) ∼ *k*_*e*_*z*. In the intermediate velocity range, viscosity contributes increasing the reaction force with the velocity, in a non-linear way, until reaction force reaches the plateau *F*(*ν* → ∞) ∼ (*k*_*e*_ + *K*)*z*. The experimental sigmoidal behavior shown in Fig 3b, which starts form a lower plateau value and then increase in a nonlinear fashion up to reach a higher plateau value, fits very well—form a qualitative point of view—the SLS model. This evidence demonstrates that ILM does not behave like an elastic body; conversely, its biomechanical response appears to be influenced by dissipative forces.

The contribution of viscous forces can be evaluated measuring the AFM hysteresis *H*, or the energy dissipated during the deformation process in a force distance cycle (Eq 2).

In Fig 3(e)–3(h) we show four representative hysteresis maps acquired on the vitreal side (e,g) and the retinal side (f,h) of a patient diagnosed with a macular pucker and a macular hole, respectively. The same colour scale was adopted to facilitate the comparison between the different samples. In Fig 3i, we show the average H value with the corresponding SEM evaluated on all the measured samples. We obtained H = 0.39±0.03 and H = 0.29±0.05 for the vitreal side (black) and H = 0.51±0.04 H = 0.47±0.03 for the retinal side (green), for macular hole and pucker, respectively. As a general comment, the retinal side shows a higher hysteresis than the vitreal one, independently of the pathology. No statistically significant difference can be detected between the two pathologies (Table 1). This finding is interesting if compared to what is shown in Fig 2m for the average Young’s modulus. According to these data, the difference in the material properties of the ILM seems to involve mainly the elastic responders of the membrane, such as collagen IV, rather than dissipative responders, like proteoglycans.

### 1.4 Morphological characterization of the ILM suggests that membranes in macular pucker are slightly rougher than in macular hole

**Fig 4:**
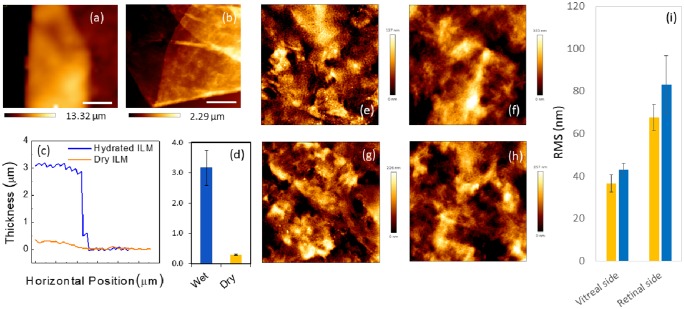
Zero force topography of a representative hydrated inner limiting membrane (ILM) specimen (a). Representative topography of a dehydrated ILM specimen acquired in contact mode (b). Representative line profiles of a hydrated (blue continuous line) and de-hydrated (orange continuous line) ILM specimen (c). Bar plot of the average ILM thickness in the hydrated and de-hydrated states (d). AFM images of the surface morphology of the vitreal side (e, f) and the retinal side (g, h) of an ILM specimen from a macular hole (e, g) and macular pucker (f, h). Scanned area 20 × 20 μm^2^. Bar plot of the mean RMS parameter for each class of tissues (i).

After mechanical measurements, the same samples underwent morphological characterization, both, in the hydrated and dehydrated state. A representative zero force-image of a hydrated sample is shown in Fig 4a. The membrane is a few microns thick and curls up towards its retinal surface because of the different stiffness of two membrane side (Fig 2m, Table 1). In Fig 4b, we show a typical AFM topography acquired in contact mode after sample dehydration. Two representative line profiles are reported in Fig 4c. A dramatic shrinkage of the membrane volume can be clearly observed after the dehydration process, confirming the high water content of the ILM. Such a high water content is the main responsible for the viscoelastic and dissipative behavior shown in Fig 3.

Measuring all the collected tissues, we obtained an average ILM thickness of (3.17 ± 0.58) *μ*m for hydrated samples and (290 ± 15) nm for dehydrated samples (Fig 4d), in agreement with previous AFM and TEM studies [37]. Morphological features of ILM specimens in the two macular pathologies were compared acquiring 20 *μ*m × 20 *μ*m (512 × 512 pixels) topographies of both membrane sides. Four representative AFM images of the retinal and vitreal side of an ILM obtained after macular hole and pucker surgery are reported in Fig 4(e)–4(h). As expected the vitreal side appears smoother that the retinal one. This is particularly relevant for AFM studies because the high corrugation of the retinal side provides an effective means to distinguish the two membrane sides in a label-free fashion. Quantitative information on the surface morphology of the samples can be obtained computing the RMS roughness (*σ*). An average *σ* of 36 ± 4 nm and 43 ± 3 nm was estimated for the vitreal side in the case of macular hole and pucker, respectively; an average *σ* of 67 ± 6 nm and 83 ± 13 nm was measured for the retinal side for macular hole and pucker (Fig 4i). The obtained values are consistent with that shown in previous experimental AFM studies [37]. Although we do not detect statistically significant difference between the two pathologies, ILM specimens extracted from patients diagnosed with macular pucker show a slightly higher roughness with respect to macular hole, for both the vitreal and retinal side.

## Discussion

In this study, we employed Atomic Force Microscopy to elucidate the mechanical and microstructural differences of internal limiting membranes extracted form patients diagnosed with macular hole and pucker. Both retinal and vitreal side of the ILM were characterized. The retinal surface of all the examined specimens appeared to be significantly stiffer than the vitreal counterpart. We measured a mean retinal-to-vitreal *E* ratio of 4.3 ± 1.8 and 9.1 ± 2.1 for macular pucker and hole, respectively. Similar results can be found in previous AFM studies. Henrich et al. reported an overall mean stiffness of 224.6 ± 43.2 kPa for the retinal side and of 44 ± 4 kPa for the vitreal side, resulting in a ratio of 4.0 ± 1.1 [11, 38]. Haritoglou and collaborators measured an average Young’s modulus of approximately 127 kPa and 27 kPa showing that the retinal side of the ILM is 5.1 times stiffer than the vitreal side [15, 37]. Similarly, Halfter and co-workers reported the epithelial side of a wide range of BMs, ILM included, is between two and four times stiffer than the stromal side [12, 15]. Such a difference in stiffness has been attributed to a change in the density of ECM proteins that undergoes a 20% increase moving from the vitreal to the retinal side, as demonstrated by Transmission Electron Microscopy [38].

Comparing that specimens obtained from patients diagnosed with macular hole and pucker, we found that the ILM retinal side is significantly stiffer in the former case. No statistically significant differences between the vitreal sides were detected. This difference might provide some hints on the reason why, despite the common aetiology, macular hole and pucker have different outcomes. Previous experimental works suggested that Müller cells are mechanosensitive and, thus, a modification in the ILM stiffness is likely to alter their interaction with the membrane itself. Müller cells, indeed, exert tractive forces on the ILM through the modulation of actin filaments, stress fibres and focal adhesion clusters. Such interactions were recently investigated *in-vitro* using collagen-based scaffolds of varying stiffness [39]. A stiffness increase was associated with a corresponding increase in the incorporation of actin filaments into the cytoskeleton and in the vinculin recruitment that, in its turn, stabilizes the focal adhesion promoting higher tensions. These findings suggest that, in the case of macular hole, an increased ILM stiffness could strengthen the adhesion forces between Müller cells and their ILM. Conversely, in the case of macular pucker, a reduced ILM stiffness could weaken these interactions. This dynamical balance between the material properties of the ILM and the adhesion behaviour of Müller cells may induce a modified mechanotransduction of vitreomacular tractions at the retinal level, thus contributing to the different outcome in the two pathologies. This hypothesis is supported by the ILM behaviour during surgical peeling that is more likely to undergo extensive fragmentation in the case of macular hole rather than macular pucker (Fig 1). Such an extensive fragmentation could be due to the increased interactions between the stiffened ILM and the underlying Müller cells, in the case of the macular hole. Conversely, in the case of macular pucker, the reduced adhesion between ILM and the underlying Müller cells is consistent with an easier ILM peeling, without membrane fractures.

Previous AFM studies on the ILM material properties were mainly focused on the measure of the Young’s modulus by analyzing FD curves with the Oliver and Pharr model and the Sneddon model (also adopted in the present study) [11, 13–15, 38]. In this regard, it should be noted that the former approach results in a lower *E* value than the latter one. As a general comment, it is worth stressing that both approaches assume that the sample behaves as an elastic body, neglecting the contribution of viscous and dissipative forces. This approximation cannot be considered strictly valid for the ILM, because of its high hydration state that hints at a viscous biomechanical response. Thickness measurements of ILM specimens (Fig 4) show, indeed, a dramatic shrinkage of the ILM after dehydration, demonstrating that a significant part of its volume is due to the water content. Such a high hydration state is due to the peculiar ILM composition. According to proteomic data, the predominant protein in the human ILM is a collagen-type IV, with a chain composition *α*3/*α*4/*α*5. The dominant laminin family member is laminin 521 and the most prominent proteoglycan is perlecan [12, 13, 37]. The latter one is the best candidate for water binding because of its long carbohydrate side chains, which are highly negative charged, thus binding counterions and large quantities of water [13].

The relevant role of viscous and dissipative forces in the biomechanical response of hydrated ILMs is further confirmed by Fig 3, showing a marked dependency of the Young’s modulus on the scanner velocity during indentation. To quantitatively account for the role of viscous forces, we measured the AFM hysteresis (Fig 3), showing a statistically significant difference between the vitreal and retinal side of the ILM. Conversely, we do not observe significant differences between the two pathologies (Table 1). The fact that the mean *H* value of the retinal side is higher than the one on the vitreal side could be correlated, on the one hand, to the different biochemical composition of the two sides, on the other hand to the presence of fragments of Müller cells footplates still attached to the membrane after surgical pealing. One of the reasons why ILM peeling could not result in a good post-operative vision is related to the damage of the underlying retinal nerve fibres, as well as the Müller, which might be correlated with a higher hysteresis value of the retinal side of the membrane. In this regard, the mean *H* value of the removed ILM could be a valuable and immediately measurable prognostic factor for the final vision outcome.

## Conclusion

Atomic Force Microscopy was employed to compare morphological and nano-mechanical properties of internal limiting membranes extracted from patients diagnosed with macular hole and macular pucker.

Topographical mapping of both the vitreal and the retinal side of the ILM showed no significant differences between the two pathologies, although specimens removed from the eyes with macular hole have a slightly lower RMS roughness than in the case of macular pucker.

Indentation-type atomic force experiments revealed no difference in the nano-elasticity of the ILM vitreal side in the two pathologies. Conversely, measurements unveiled that the retinal side of ILMs extracted from eyes with macular holes are significantly stiffer than in the eyes with macular pucker. Such a mechanical difference is highly interesting towards a better understanding of the mechanism behind the formation of MH and MP. The retinal side of the ILM is indeed in contact with Müller cells, a type of retinal glial cells with mechanosensitive properties that allow them to adjust their adhesion properties in response to stiffness variation. Therefore, we hypothesize that the measured difference in the ILM stiffness may be related to a different mechanotransduction of vitreomacular tractions associated with PVD, thus contributing to the different clinical outcome in the two pathologies. These results help explain the difference in the ILM behaviour during surgical peeling in the two pathologies. ILM appears to be more adherent to the retinal surface in macular hole rather than in macular pucker; moreover it undergoes extensive fragmentation in the case of macular hole, which is not observed in macular pucker. Such a strong adhesiveness and extensive fragmentation is consistent with the proposed increased interactions between the stiffened ILM and the underlying Müller cells, in the case of the macular hole. The dissipative behaviour of the membranes was also investigated measuring AFM hysteresis *H* during indentation. No statistically significant difference of the *H* values was detected, suggesting that, at the biochemical level, the difference in the material properties of ILM involves mainly elastic responders of the membrane, such as collagen IV, rather than dissipative responders, like proteoglycans. Taken together our results have the potential to stimulate the development of dedicated surgical protocols, which properly take into account the differences in the material properties of ILM in the two pathologies. Moreover, it opens up to further pharmacological treatment with drugs for maculopathies, which specifically target the focal adhesion cluster between the internal limiting membrane and the Müller cells.
